# Effect of L-carnitine on the hepatic transcript profile in piglets as animal model

**DOI:** 10.1186/1743-7075-8-76

**Published:** 2011-10-31

**Authors:** Janine Keller, Robert Ringseis, Steffen Priebe, Reinhard Guthke, Holger Kluge, Klaus Eder

**Affiliations:** 1 Institute of Animal Nutrition and Nutrition Physiology, Justus-Liebig-Universität Gießen, Heinrich-Buff-Ring 26-32, 35392 Gießen, Germany; 2 Institute of Agricultural and Nutritional Sciences, Martin-Luther-University Halle-Wittenberg, Von-Danckelmann-Platz 2, 06120 Halle (Saale), Germany; 3 Hans-Knöll-Institute, Research Group Systems Biology/Bioinformatics, Beutenbergstr. 11a, 07745 Jena, Germany

**Keywords:** carnitine, pig, microarray, gene expression, liver

## Abstract

**Background:**

Carnitine has attracted scientific interest due to several health-related effects, like protection against neurodegeneration, mitochondrial decay, and oxidative stress as well as improvement of glucose tolerance and insulin sensitivity. The mechanisms underlying most of the health-related effects of carnitine are largely unknown.

**Methods:**

To gain insight into mechanisms through which carnitine exerts its beneficial metabolic effects, we fed piglets either a control or a carnitine supplemented diet, and analysed the transcriptome in the liver.

**Results:**

Transcript profiling revealed 563 genes to be differentially expressed in liver by carnitine supplementation. Clustering analysis of the identified genes revealed that most of the top-ranked annotation term clusters were dealing with metabolic processes. Representative genes of these clusters which were significantly up-regulated by carnitine were involved in cellular fatty acid uptake, fatty acid activation, fatty acid β-oxidation, glucose uptake, and glycolysis. In contrast, genes involved in gluconeogenesis were down-regulated by carnitine. Moreover, clustering analysis identified genes involved in the insulin signaling cascade to be significantly associated with carnitine supplementation. Furthermore, clustering analysis revealed that biological processes dealing with posttranscriptional RNA processing were significantly associated with carnitine supplementation.

**Conclusion:**

The data suggest that carnitine supplementation has beneficial effects on lipid and glucose homeostasis by inducing genes involved in fatty acid catabolism and glycolysis and repressing genes involved in gluconeogenesis.

## Background

Carnitine (3-hydroxy-4-*N, N, N*-trimethylaminobutyric acid) belongs to the class of conditionally essential nutrients and has a number of indispensable functions in intermediary metabolism. Carnitine is necessary for fatty acid metabolism due to its role in the transfer of long-chain fatty acids (acyl groups) from the cytosol into the mitochondrial matrix for subsequent β-oxidation [1]. Moreover, carnitine facilitates the transport of peroxisomal β-oxidation products to the mitochondria, the export accumulating acyl-groups and acts as a CoA buffer in mammalian cells [2]. Carnitine in the body originates from intestinal absorption from dietary sources, especially meat, fish and dairy products [3] and enzyme catalyzed endogenous synthesis [4], which involves five enzymatic steps. Given that the last enzyme required for carnitine synthesis, γ-butyrobetaine hydroxylase, is only active in liver and kidney, other tissues than liver and kidney are dependent on the active uptake of carnitine from blood into tissues which is catalyzed by novel organic cation transporters (OCTN), particularly OCTN2 which is the physiologically most important carnitine transporter [5].

In livestock animal nutrition, supplementation with L-carnitine has attracted great interest due to its ability to improve performance characteristics, such as growth rate, feed conversion ratio, protein:fat accretion [6-8]. However, L-carnitine is also of interest for human nutrition because recent studies indicated that L-carnitine exerts several other effects which may be useful for the treatment of degenerative and metabolic disorders. For instance, supplementation of L-carnitine or acyl-carnitines has been associated with protecting against neurodegeneration [9], age-dependent mitochondrial decay [10], and oxidative stress [11]. In addition, L-carnitine was shown to improve glucose tolerance and insulin sensitivity in animals as well as in healthy and diabetic patients [12,13]. Moreover, L-carnitine supplementation was shown to be useful for the treatment of hepatic steatosis induced by total parenteral nutrition in rodents [14] and nonalcoholic steatohepatitis in humans [15,16]. Furthermore, L-carnitine was reported to reduce hepatic inflammation and plasma levels of cytokines and acute phase proteins in patients with chronic hepatitis C [16]. The mechanisms underlying many of these beneficial effects of L-carnitine are largely unknown. Therefore, the present study was designed to gain insight into mechanisms and pathways influenced by L-carnitine. For this end, we used piglets, indicating many genetical and physiological similarities with humans, making it an optimal species to study the effect of L-carnitine on transcript profile.

We considered the liver as the target organ because it plays a central role in whole body metabolism by regulating glucose and lipid homeostasis as well as protein synthesis. In this sense, we used liver samples taken from a previous experiment with piglets [17] which were fed a either a control diet with a low native carnitine content or a diet supplemented with 500 mg/kg diet L-carnitine and performed genome-wide transcript profiling in the liver of these piglets.

## Methods

### Animal experiment

The animal experiment was approved by the local Animal Care and Use Committee. As described recently in more detail [17], the experiment was performed with sixteen male crossbred pigs [(German Landrace × Large White) × Pietrain] with an average body weight of 10 ± 1 (mean ± SD) kg. The pigs were assigned to two groups (control group and carnitine group) and fed experimental diets for a period of 21 days. The control group received a basal diet with a low native carnitine concentration (< 5 mg/kg) which was nutritionally adequate for growing pigs in a body weight range between 10 and 20 kg, according to the recommendations of the German Society for Nutrition Physiology (Gesellschaft für Ernährungsphysiologie, 2006). The carnitine group received the same diet supplemented with 500 mg L-carnitine (obtained from Lohmann Animal Health, Cuxhaven, Germany) per kg. Blood was collected and plasma obtained by centrifugation of the blood, and liver was excised. Plasma and liver samples were immediately stored at -80°C until analysis. A full description of diet composition, feeding regime, sample collection and carnitine analysis of diets and tissues can be found in our recent publication [17].

### Carnitine analysis

Concentrations of free carnitine, acetyl carnitine and propionyl carnitine in liver of pigs fed either a control diet or a diet supplemented with 500 mg/kg carnitine for 20 days were determined by tandem mass spectrometry using deuterated carnitine-d_3 _(Larodane Fine Chemicals, Malmö, Sweden) as internal standard as described recently in detail [17]. Concentration of total carnitine represents the sum of free carnitine, acetyl carnitine and propionyl carnitine.

### RNA isolation and quality control

Total RNA was prepared from 20-30 mg of frozen liver tissue using the RNeasy Minikit (Qiagen, Hilden, Germany) according to the manufacturer's protocol. Afterwards, the RNA concentration and purity were estimated from the optical density at 260 and 280 nm, respectively. The A260/280 ratio of all individual samples was 1.98 ± 0.02 (mean ± SD). The integrity of the total RNA was checked by 1% agarose gel electrophoresis. RNA was judged as suitable for array hybridization only if the samples exhibited intact bands corresponding to the 18S and 28S ribosomal RNA subunits.

### Microarray analysis

For microarray analyses, two RNA pools for each group (control, n = 2; carnitine group, n = 2) were prepared, with RNA from 4 animals contributing to each RNA pool. Hybridization to the Affymetrix GeneChip porcine genome arrays (Affymetrix) containing 23,937 probe sets that represent approximately 23,256 porcine transcripts and quality assessment of the hybridization process were performed at the Center of Excellence for Fluorescent Bioanalytics (KFB) at the University of Regensburg for hybridization to the Affymetrix GeneChip porcine genome arrays (Affymetrix, UK). The microarray data related to all samples have been deposited in NCBI's Gene Expression Omnibus (GEO) public repository (GEO accession number GSE22931) [18]. Data analyses and functional interpretation of microarray data using the bioinformatic tools from the Database for Annotation, Visualization and Integrated Discovery (DAVID) bioinformatic resource [19] were performed as described recently in detail [17].

### Quantitative real-time RT-PCR (qPCR)

Differential expression data of selected genes, FbxL3 (F-box/LRR-repeat protein 3), FbxL20 (F-box/LRR-repeat protein 20), FbxO32 (F-box protein 32), ESRRG (estrogen-related receptor gamma isoform 2), HECTD2 (HECT domain containing 2 isoform b), DRE1 (DRE1 protein), GPD1 (Glycerol-3-phosphate dehydrogenase), MTTP (Microsomal triglyceride transfer protein), ACSL3 (Long-chain-fatty-acid-CoA ligase 3), ACADSB (Acyl-CoA dehydrogenase, short/branched chain specific), GLUT8 (Glucose transporter type 8), GCK (Glucokinase), GPAT (Glycerol-3-phosphate acyltransferase) and USP10 (ubiquitin specific peptidase 10) obtained from Affymetrix GeneChip analysis were validated by using qPCR carried out on a Rotorgene 2000 system (Corbett Research, Mortlake, Australia). For qPCR all individual samples (n = 8/group) contributing to the RNA pools for microarray analysis were used. cDNA synthesis and qPCR analysis was performed as described recently in detail [17]. Gene-specific primers (Eurofins MWG Operon, Ebersberg, Germany) were designed using Primer3 and BLAST. Characteristics of the porcine primer pairs are listed in Table 1. Expression values of selected genes were normalized using the GeNorm normalization factor. Procedure of normalization, characteristics of gene-specific primers and the average expression stability ranking of the six potential reference genes in liver of piglets were previously described in detail [20]. Relative expression ratios are expressed as fold changes of mRNA abundance in the carnitine group compared to the control group.

**Table 1:** Characteristics of porcine primer pairs used for validation of microarray analysis using RT-PCR

Gene symbol	Primer sequence (5^'^-3^'^)	GenBank **accession no**.	Product size (bp)
ACADSB	For:TCGTGATACCGAGGGCCTCCG	XM_001926297.2	196
	Rev:TCCCAGCATCTGTGCCGCAA		
ACSL3	For:TCGCTGCACAGGCCTGCTTC	NM_001143698.1	174
	Rev:GCAGGCGCGGCACTAGAGAG		
DRE1	For:CAACAACCTCCGATACTACC	NP_060114.2	158
	Rev:GGTCCTCCACCAATCACAAA		
ESRRG	For:GGATCAGATGAGTCTTCTGC	XM_003357621.1	127
	Rev:GGACTGGTCTTCATCCATTAT		
FbxL3	For:CATAGGAGACACACCGTCTA	Q9UKT7	637
	Rev:GTGGGCATCATGTCTGGAAA		
FbxL20	For:GTGAGGGATGTCCACTGTTG	XM_003131523.2	128
	Rev:CTGTGTGCAGCCTTTTAAGAA		
FbxO32	For:TCACAGCTCACATCCCTGAG	NM_001044588	167
	Rev:GACTTGCCGACTCTCTGGAC		
GCK	For:GAGCGAGAGAGCAGAGCCTCAGA	XM_003356680.1	221
	Rev:CTGGAGCCAGCCTCCGAACG		
GLUT8	For:GTGGAGCCCACCGATGCCAG	EU012361.2	145
	Rev:CCACGCCCTTGACGTGCAGA		
GPAT	For:GAATTGATCTCTCCACGTTG	XM_001927875.1	257
	Rev:CCTCCATGATAAAGTCGTGG		
GPD1	For:GGCCGGCTGGCACACTTTGA	NM_001190240.1	354
	Rev:CATGGGGATGCCAAGGCGCT		
HECTD2	For:GGTTTGGACAGAGGATCCAAA	XM_003361201.1	130
	Rev:CATTCTTGATGTTAGGGAAAAC		
MTTP	For:TCCCGCTGCACCAAGAGAACT	NM_214185.1	151
	Rev:TACCTCGGCACGGTGCATCGT		
USP10	For:GTGGTGTACCAGCAGAGCT	XM_003126825.2	157
	Rev:GCTTGGTTTTGGTGGTGTAG		

### Statistical analysis

Values presented in the text are means ± SD. Data were analyzed by one factorial analysis of variance with dietary carnitine concentration as factor using the Minitab statistical software (Release 13, Minitab Inc., State College, PA, USA). For statistical significant *F*-values (P < 0.05), means of both groups were compared by Fisher's multiple range test.

## Results

### Feed intake, final body weight and feed conversion ratio

Feed intake, final body weights and feed conversion ratio of piglets were not different between both groups [17].

### Concentration of carnitine in the liver

**Figure 1:**
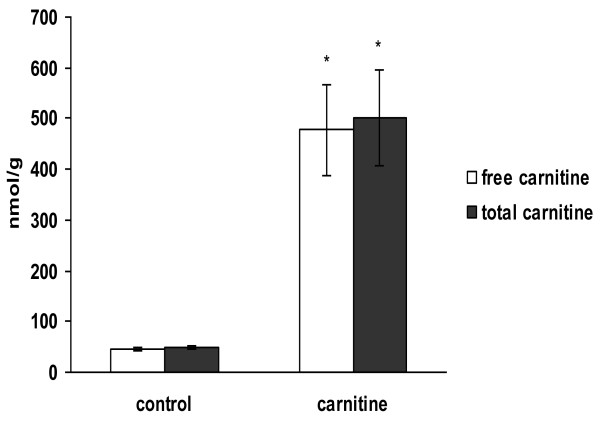
**Concentrations of free and total carnitine in the liver of growing piglets fed either a control diet or a diet supplemented with 500 mg L-carnitine per kg diet for 21 days**. Bars are mean ± SD (n = 8/group), * indicates significant differences (P < 0.05) between groups (Fisher's multiple range test).

Pigs supplemented with L-carnitine had approximately 10-fold higher concentrations of free and total carnitine in the liver (P < 0.05; Figure 1) than control pigs.

### Identification of differentially expressed genes

**Figure 2:**
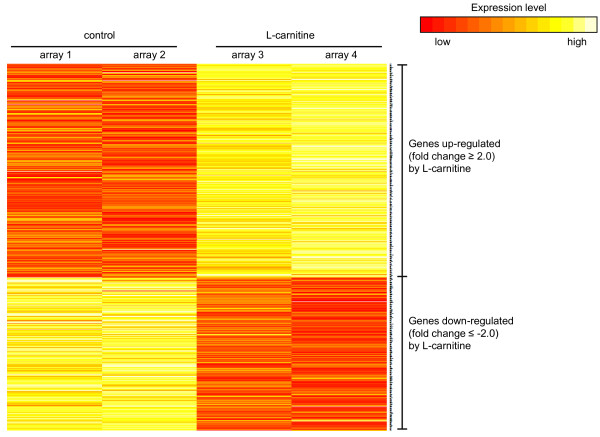
**Heat map illustrating the level of expression for the differentially expressed genes identified as a response to feeding a diet containing 500 mg L-carnitine per kg diet**. Based on log2 transformed signal intensities the heat map was generated with software package R (URL: http://www.R-project.org).

A total of 638 probe sets were differentially expressed (fold change ≥ 2.0 and ≤ -2.0) between the L-carnitine and the control group. Of these probe sets, 372 were up-regulated by L-carnitine and 266 probe sets were down-regulated by L-carnitine. The 15 most strongly up-regulated and down-regulated probe sets are shown in Table 2. Because the Affymetrix GeneChip porcine genome array is poorly annotated, the differentially expressed probe sets were largely annotated by the annotation list created by Tsai et al. [21]. 563 porcine Affymetrix probe sets were matched to human RefSeq entries and converted into human Affymetrix probeset IDs. Conversion of porcine Affymetrix probe set IDs into the human probe set IDs was necessary for subsequent analysis by the DAVID bioinformatic resource because this platform cannot use porcine gene information. The distribution of signal intensities of the differentially expressed probe sets of the two control and carnitine arrays are shown in Figure 2.

**Table 2:** The 15 most strongly up- and down-regulated genes in the liver of growing piglets fed with or without L-carnitine

Probe set ID	Gene name (Gene symbol)	FC*	Probe set ID	Gene name (Gene symbol)	FC*
*Up-regulated genes *			*Down-regulated genes*		
Ssc.16377.2.A1_at	Glutathione S-transferase A3-3 (GSTA3)	129.6	Ssc.27111.1.A1_at	Kelch-like protein 8 (KLHL8)	-7.9
Ssc.18484.1.S1_at	Hexokinase D (GCK)	26.5	Ssc.30350.1.A1_at	Homeobox protein Meis1 (MEIS1)	-6.6
Ssc.14503.1.S1_at	Apolipoprotein A-IV precursor (ApoA4)	16.6	Ssc.15845.1.S1_at	Mannose-binding protein C precursor (MBP-C)	-5.8
Ssc.13302.1.A1_at	Sentrin-specific protease 6 (SENP6)	13.0	Ssc.451.1.A1_at	Insulin-like growth factor binding protein 1 precursor (IGFBP-1)	-5.8
Ssc.12965.1.A1_at	Sprouty homolog 3 (SPRY3)	11.8	Ssc.22959.1.S1_at	Phosphoenolpyruvate carboxykinase, cytosolic (PCK1)	-5.7
Ssc.30459.1.A1_at	R3H domain protein 1 (R3HDM)	11.3	Ssc.24758.1.A1_at	estrogen-related receptor gamma isoform 2 (ESRRG)	-5.3
Ssc.25850.1.A1_at	Telomerase-binding protein p23 (TEPB)	9.2	Ssc.21169.1.S1_at	Synaptic vesicular amine transporter (SLC18A2)	-5.1
Ssc.5327.2.A1_at	Cytochrome P450 2J2 (CYP2J2)	9.2	Ssc.20502.1.S1_at	Serine/threonine-protein kinase (ULK1)	-5.0
Ssc.9177.1.A1_at	SPARC related modular calcium-binding protein 1 precursor (SMOC1)	8.5	Ssc.29392.1.A1_at	DRE1 protein (DRE1)	-4.7
Ssc.30207.1.A1_at	Ubiquitin carboxyl-terminal hydrolase 1 (USP1)	8.3	Ssc.14386.1.A1_at	Cyclin G2 (CCNG2)	-4.7
Ssc.8700.1.A1_at	Heterogeneous nuclear ribonucleoprotein M (hnRNP M)	8.1	Ssc.29946.1.A1_at	T-cell lymphoma breakpoint-associated target 1 (TCBA1)	-4.2
Ssc.8308.1.A1_at	cell adhesion molecule with homology to L1CAM precursor (CHL1)	7.6	Ssc.28087.1.A1_at	oxidation resistance 1 (OXR1)	-4.2
Ssc.18681.1.A1_at	Metabotropic glutamate receptor 5 precursor GRM5)	7.5	Ssc.2274.1.A1_at	Ephrin-A1 precursor (EFNA1)	-4.1
Ssc.29205.1.A1_at	Serine/threonine-protein kinase (Nek7)	7.3	Ssc.13343.1.A1_at	CD109 (CD109)	-4.1
Ssc.18831.1.A1_at	Glutaminase, kidney isoform, mitochondrial precursor (GLS)	6.8	Ssc.30210.1.A1_at	Testican-1 precursor (SPOCK)	-4.0

* FC = fold change

### Real-time RT-PCR verification of microarray data

A total of 14 genes were selected to validate the microarray data by the use of real-time RT-PCR. As shown in Table 3, all tested genes were differentially expressed with microarray analysis, however 3 genes were not significant at an FDR *P *value < 0.05. The magnitude of differential expression tended to be higher by qPCR than those obtained from microarray analysis.

**Table 3:** qPCR and microarray gene expression analyses of liver tissue

Gene symbol	Mean fold changes	P-value	
	Microarray	qPCR	qPCR
ACADSB	2.24	4.61	0.057
ACSL3	2.15	3.48	0.001
DRE1	-4.8	-3.3	0.018
ESRRG	-5.6	-12.5	0.003
FbxL20	-2.9	-14.3	0.002
FbxL3	-2.4	-4.4	0.026
FbxO32	-3.6	-3.4	0.009
GCK	26.53	1.97	0.107
GLUT8	3.55	4.55	0.018
GPAT	-2.84	-1.67	0.173
GPD1	2.87	3.11	0.031
HECTD2	-2.9	-4.6	0.004
MTTP	2.14	1.75	0.031
USP10	-2.2	-3.2	0.026

### Identification of overrepresented annotation terms

563 genes identified to be differentially expressed were used for gene-term enrichment analysis using the DAVID Functional Annotation Chart tool. The analyses were based on the GO category biological process. The GO analysis assigned the 563 differentially expressed genes to 21 biological processes (p-value < 0.01). Most genes were allocated to the annotation terms cellular process (357 genes), metabolic process (262 genes), and cellular metabolic process (239 genes). The most significantly enriched annotation terms were (top-ranked: lowest p-values): cellular process (P = 2.0E-04), ribosome biogenesis (P = 3.8E-04), developmental process (P = 4.0E-04), ribonucleoprotein complex biogenesis (P = 4.6E-04), cellular metabolic process (P = 6.5E-04), multicellular organismal development (P = 1.5E-03), cellular protein metabolic process (P = 1.9E-03), rRNA processing (P = 2.1E-03), and rRNA metabolic process (P = 2.8E-03). The annotation terms with the highest fold enrichment were triglyceride metabolic process (4.5-fold), rRNA processing (3.5-fold), ribosome biogenesis (3.4-fold), and rRNA metabolic process (3.4-fold).

### Identification of clusters of functionally related annotation terms

To identify clusters of functionally related biological processes we used the DAVID functional annotation clustering tool. Clusters were ranked according to the enrichment score for each cluster reflecting the geometric mean of all the enrichment p-values (EASE scores) of each annotation term in the cluster. The 10 top-ranked clusters showing the highest enrichment scores are shown in Table 4. The top-ranked clusters allocated annotation terms dealing with RNA splicing (cluster 1), ribosomal RNA processing and non-coding RNA processing (cluster 2), triglyceride, acylglycerol, neutral lipid and glycerol ether metabolism (clusters 3 and 4) and regulation of glucose import and glucose transport (cluster 5). Further clusters summarized annotation terms dealing with chromosome localization (cluster 6), posttranslational protein folding (cluster 7), carbohydrate biosynthetic processes (cluster 8) and organic acid metabolic processes (cluster 9), and modification-dependent protein catabolic process and proteolysis involved in cellular protein catabolic process (cluster 10).

**Table 4:** Identification of functionally related annotation groups (GO category biological process)

Cluster	GO terms	P-value
1	RNA splicing, via transesterification reactions	7.8E-03
	RNA splicing, via transesterification reactions with bulged adenosine as nucleophile	7.8E-03
	nuclear mRNA splicing, via spliceosome	7.8E-03
		
2	ribosomal RNA processing	2.1E-03
	ribosomal RNA metabolic process	2.8E-03
	non-coding RNA processing	1.2E-01
		
3	triglyceride metabolic process	9.9E-03
	acylglycerol metabolic process	1.7E-02
	neutral lipid metabolic process	1.8E-02
	glycerol ether metabolic process	2.0E-02
	organic ether metabolic process	2.3E-02
		
4	triglyceride biosynthetic process	3.0E-02
	neutral lipid biosynthetic process	5.1E-02
	acylglycerol biosynthetic process	5.1E-02
	glycerol ether biosynthetic process	6.0E-02
		
5	positive regulation of glucose import	2.9E-02
	positive regulation of glucose transport	2.9E-02
	regulation of glucose import	8.0E-02
	regulation of glucose transport	8.6E-02
		
6	mitotic metaphase plate congression	2.9E-02
	metaphase plate congression	4.3E-02
	chromosome localization	7.6E-02
	Establishment of chromosome localization	7.6E-02
		
7	chaperone mediated protein folding requiring cofactor	5.3E-02
	'de novo' posttranslational protein folding	5.3E-02
	'de novo' protein folding	5.3E-02
		
8	hexose biosynthetic process	7.1E-01
	monosaccharide biosynthetic process	7.1E-01
	alcohol biosynthetic process	7.1E-01
	cellular carbohydrate biosynthetic process	7.1E-01
	carbohydrate biosynthetic process	7.1E-01
		
9	carboxylic acid metabolic process	1.9E-01
	oxoacid metabolic process	1.9E-01
	organic acid metabolic process	2.0E-01
		
10	modification-dependent protein catabolic process	2.4E-01
	modification-dependent macromolecule catabolic process	2.4E-01
	proteolysis involved in cellular protein catabolic process	3.0E-01
	cellular protein catabolic process	3.1E-01
	protein catabolic process	3.7E-01

## Discussion

In the present study we aimed to get insight into potential mechanisms of L-carnitine by applying genome-wide transcript profiling in the liver of piglets. After feeding carnitine supplemented diets for 3 weeks, concentrations of free and total carnitine in the liver of the piglets were markedly increased (approximately 10-fold) compared to piglets fed diets without supplemental L-carnitine, indicating that the supplemental L-carnitine significantly improved the carnitine status of the piglets. As a main result we observed that 563 genes were differentially expressed by L-carnitine. This shows that supplemental L-carnitine influences gene expression in the liver of piglets and indicates that at least some of the biological effects of L-carnitine are mediated by altering gene transcription. To extract biological meaning from the observed alterations in gene expression we performed gene term enrichment analysis and functional clustering analysis with the 563 differentially expressed genes. Gene term enrichment analysis revealed that the most frequent biological processes associated with L-carnitine supplementation were dealing with metabolic processes. This was not surprising considering that the main function of L-carnitine is to stimulate energy metabolism by acting as shuttling molecule for long-chain fatty acids which also enhances the metabolic flux of glucose through the glycolytic chain. This was also confirmed by clustering analysis showing that 6 out of the 10 top-ranked clusters were dealing with metabolic processes. Representative genes from one of these clusters dealing with metabolic processes (carboxylic acid metabolic process, oxoacid metabolic process, organic acid metabolic process) encoded proteins or enzymes involved in cellular fatty acid uptake (SLC27A6, solute carrier family 27/fatty acid transporter, member 6), fatty acid activation (ACSL3, Long-chain-fatty-acid-CoA ligase 3) and fatty acid β-oxidation (ACADSB, Acyl-CoA dehydrogenase, short/branched chain specific), and most of these genes including SLC27A6, ACSL3 and ACADSB were found to be significantly up-regulated by L-carnitine supplementation by microarray analysis and confirmed by qPCR, a more sensitive method for gene expression analysis. Thus, our data indicate that the well-known stimulatory effect of carnitine on fatty acid β-oxidation [22,23] is at least partially mediated by stimulating the transcription of genes involved in cellular fatty acid uptake, fatty acid activation and β-oxidation.

Clustering analysis further revealed that L-carnitine supplementation was significantly associated with biological processes involved in glucose metabolism, like glucose transport, conversion of glucose into glucose 6-phosphate, and glycolysis, and hexose biosynthetic processes, like gluconeogenesis. Representative genes included GLUT8 (glucose transporter type 8), GCK (Hexokinase D), GPD1 (Glycerol-3-phosphate dehydrogenase), PCK1 (Phosphoenolpyruvate carboxykinase), and FBP2 (Fructose-1,6-bisphosphatase isozyme 2). Moreover, the tandem enzyme PFKFB3 (6-phosphofructo-2-kinase/fructose-2,6-biphosphatase 3) which is responsible for maintaining the cellular levels of fructose-2,6-biphosphate, the most potent allosteric activator of one of the key regulatory enzymes of glycolysis, 6-phosphofructo-1-kinase, was also identified to be differentially expressed by L-carnitine. All the genes dealing with glucose metabolism like GLUT8, GCK and GPD1 were markedly up-regulated, at least 4-fold, by the supplemental L-carnitine. These strongly up-regulation could also observed by qPCR, supporting microarray analysis. GCK which is the predominant hexokinase isoenzyme in the liver phosphorylating glucose for subsequent metabolism by either glycolysis, pentose phosphate shunt or glycogen synthesis was even induced 27-fold by L-carnitine supplementation - indicating that L-carnitine has a dramatic effect on glucose metabolism. Several studies have already shown that carnitine supplementation increases glucose disposal and glucose oxidation in animals as well as in healthy and diabetic patients [12,24,25] due to activation of the pyruvate dehydrogenase complex [26]. Besides allosteric regulation of the activity of enzymes of the glycolytic pathway, it is also well known that the flux through the glycolytic pathway can be increased by induction of the rate-limiting enzymes of this pathway, GCK and PFK1 (6-phosphofructo-1-kinase). Thus, our observations suggest that up-regulation of genes involved in glucose uptake, glucose phosphorylation and glycolysis also contributes to increased glucose oxidation by supplemental L-carnitine. In contrast to the genes involved in glucose metabolism, genes involved in gluconeogenesis, like PCK1 and FBP2 were significantly down-regulated in the liver of piglets by L-carnitine supplementation. This indicates that the positive effect of carnitine on glucose utilization is explained not only by stimulation of glycolysis but also suppression of gluconeogenesis in the liver. It has been recently shown in rats that dietary L-carnitine is capable of restoring an increase in the activity of the gluconeogenic enzymes PCK1, FBP2 and glucose 6-phosphatase caused by feeding fructose [27], which increases the availability of the gluconeogenic substrates pyruvate, lactate and glycerol. The present findings suggest that inhibition of transcription of gluconeogenic genes by L-carnitine also contributes to the suppression of gluconeogenesis by carnitine, which has not been demonstrated yet. The exact reason for this effect of L-carnitine remains to be established. However, it has been suggested that the inhibitory effect of L-carnitine on gluconeogenic enzyme activities is the consequence of an improvement in the action of insulin [12,28], which is a known repressor of expression of gluconeogenic genes. Supportive of this assumption is also the identification of another annotation term cluster dealing with positive regulation of glucose import. Considering that insulin is the most important regulator of glucose import, it was not surprising that genes belonging to this cluster were involved in insulin signaling, like IRS2 (Insulin receptor substrate-2), PIK3R1 (Phosphatidylinositol 3-kinase regulatory alpha subunit), and ERBB3 (Receptor protein-tyrosine kinase erbB-3 precursor). Besides regulation by insulin action, key gluconeogenic enzymes such as FBP2 are also known to be subject to complex allosteric regulation. Noteworthy, one of the allosteric inhibitors of FBP2 is fructose-2,6-biphosphate whose cellular levels are controlled by the abovementioned tandem enzyme PFKFB3. Thus, the activity of gluconeogenic enzymes may be influenced by L-carnitine through regulating the cellular availability of allosteric enzyme regulators as well.

Two other identified annotation term clusters were dealing with triglyceride metabolic and triglyceride biosynthetic processes. Representative genes were GPAT, which esterifies acyl-groups from acyl-ACP to the sn-1 position of glycerol-3-phosphate, an essential step in glycerolipid biosynthesis, and MTTP, which catalyses the transport of triglyceride, cholesteryl ester, and phospholipid between phospholipid surfaces, and is required for the secretion of apolipoprotein B containing lipoproteins from the liver. In rodents, L-carnitine administration was demonstrated to decrease liver lipids and hepatic steatosis after administration of a high fat diet, after parenteral nutrition, or after alcohol intoxication [14,29,30], with the mechanisms of action being largely unknown. Our observations that GPAT was down-regulated whereas MTTP and genes involved in fatty acid catabolism (SLC27A6, ACSL3, ACADSB) were up-regulated by L-carnitine supplementation in the liver of the piglets, confirmed by qPCR. Thus, our data indicates that inhibition of glycerolipid biosynthesis and stimulation of lipoprotein secretion and fatty acid catabolism may contribute to the decreased liver lipids in rodents fed L-carnitine.

Besides metabolic processes, clustering analysis revealed that biological processes dealing with posttranscriptional RNA processing (mRNA splicing, ribosomal RNA processing, non-coding RNA processing) were significantly associated with L-carnitine supplementation. Noteworthy, almost all of these genes were significantly up-regulated in the liver of the piglets by the supplemental L-carnitine indicating that the biological functions exerted by the encoded proteins are stimulated by L-carnitine. Posttranscriptional RNA processing, which includes precursor-mRNA splicing, is one of the main regulatory mechanisms of gene expression which results in a repertoire of mRNAs, and consequently of proteins, much larger than expected from the number of genes. This process contributes substantially to cell-specific and tissue-specific gene expression, and it is estimated that over 60% of human genes are alternatively spliced [31]. Although the exact biological meaning of the up-regulation of genes dealing with posttranscriptional RNA processing by L-carnitine is unclear, it is obvious that L-carnitine supplementation has a stimulatory effect on this important regulatory mechanism of gene expression. It is therefore possible that the alterations in gene expression observed with L-carnitine supplementation are mediated, at least partially, by modulating posttranscriptional RNA processing.

As far as the physiological relevance of the carnitine dose (500 mg/kg) used in this study is concerned, it has to be noted that omnivorous humans are reported to generally ingest 0.3-1.9 mg of carnitine per day and kg of body weight [32]. Based on a daily feed consumption of approximately 500 g per day and a final body weight of the piglets of about 17 kg this relates to 15 mg of carnitine/kg body weight indicating that the carnitine dose applied to the piglets was at least 8-fold higher than achieved in humans by a normal diet. However, when compared to several clinical trials in which free carnitine was supplemented up to 3 g per day and subject [33-35], which relates to 40 mg carnitine/kg body weight for a human weighing 70 kg, the dose applied in our study can be regarded as low. Therefore, the beneficial effects of carnitine supplementation on several metabolic parameters observed in the abovementioned clinical trials may be at least partially explained by the alterations in gene expression found in our pig model. In several clinical trials, carnitine supplementation was shown to improve glucose homeostasis and insulin sensitivity in obese, insulin resistant and diabetic subjects [36,37]. Due to theses effects carnitine supplementation has gained significant attention as a tool for the treatment or prevention of insulin resistance and type 2 diabetes mellitus [38]. The improvement of glucose tolerance has been explained by a normalization of mitochondrial fuel metabolism, which is perturbed during insulin resistance due to an intracellular accumulation of acyl-CoA derivatives. Recent evidence indicates that carnitine supplementation corrects these mitochondrial perturbations through an increased efflux of acyl-carnitine out of mitochondria and cells into the blood [39]. Based on our transcriptomic data it cannot be evaluated whether or not acyl-carnitine efflux and mitochondrial function was stimulated by carnitine in the piglets of this study. However, since convincing evidence from both human and animal studies suggests that carnitine lowers "mitochondrial stress" and improves mitochondrial function, in particular in the face of energy surplus, we suggest that carnitine also improved mitochondrial function in our pig model.

In conclusion, results of the present study show that supplemental L-carnitine influences gene expression in the liver of growing piglets. Our data suggest that L-carnitine supplementation has beneficial effects on lipid and glucose homeostasis by inducing genes involved in fatty acid catabolism and glycolysis and repressing genes involved in gluconeogenesis. In addition, the data indicate that the effects of L-carnitine on transcription of glycolytic and gluconeogenic genes are mediated by potentiating the action of insulin and that at least some of the alterations in gene expression observed with L-carnitine supplementation are mediated by modulating posttranscriptional RNA processing. Regarding the strong similarities between pigs and humans with regard to metabolism, our data obtained in piglets can be considered as relevant for humans. Future studies employing both, transcriptomics and metabolomics are required to confirm a correlation between alterations in gene expression and modulation of carbon fluxes through metabolic pathways.

## Competing interests

The authors declare that they have no competing interests.

## Authors' contributions

RR and KE designed research and coordinated the study; JK carried out the feeding experiment and the molecular biological analyses; HK supervised the feeding experiment and performed the chemical analysis of experimental diets; JK and RR performed bioinformatical data analyses; SP and RG were involved in microchip data visualization; JK, RR and KE wrote the paper. KE had primary responsibility for final content. All authors read and approved the final manuscript.
